# Deep Learning for Activity Recognition in Older People Using a Pocket-Worn Smartphone

**DOI:** 10.3390/s20247195

**Published:** 2020-12-15

**Authors:** Yashi Nan, Nigel H. Lovell, Stephen J. Redmond, Kejia Wang, Kim Delbaere, Kimberley S. van Schooten

**Affiliations:** 1 Graduate School of Biomedical Engineering, University of New South Wales, Sydney 2033, Australia; nanyashi1996@gmail.com (Y.N.); n.lovell@unsw.edu.au (N.H.L.); stephen.redmond@ucd.ie (S.J.R.); kejia.wang@gmail.com (K.W.); 2 Neuroscience Research Australia, University of New South Wales, Sydney 2031, Australia; k.delbaere@neura.edu.au; 3 School of Electrical and Electronic Engineering, University College Dublin, Dublin, Ireland; 4 School of Public Health and Community Medicine, University of New South Wales, Sydney 2052, Australia

**Keywords:** activity recognition, accelerometry data, deep learning, older people, smartphone

## Abstract

Activity recognition can provide useful information about an older individual’s activity level and encourage older people to become more active to live longer in good health. This study aimed to develop an activity recognition algorithm for smartphone accelerometry data of older people. Deep learning algorithms, including convolutional neural network (CNN) and long short-term memory (LSTM), were evaluated in this study. Smartphone accelerometry data of free-living activities, performed by 53 older people (83.8 ± 3.8 years; 38 male) under standardized circumstances, were classified into lying, sitting, standing, transition, walking, walking upstairs, and walking downstairs. A 1D CNN, a multichannel CNN, a CNN-LSTM, and a multichannel CNN-LSTM model were tested. The models were compared on accuracy and computational efficiency. Results show that the multichannel CNN-LSTM model achieved the best classification results, with an 81.1% accuracy and an acceptable model and time complexity. Specifically, the accuracy was 67.0% for lying, 70.7% for sitting, 88.4% for standing, 78.2% for transitions, 88.7% for walking, 65.7% for walking downstairs, and 68.7% for walking upstairs. The findings indicated that the multichannel CNN-LSTM model was feasible for smartphone-based activity recognition in older people.

## 1. Introduction

Being physically active is critical for older people to reduce their risk of developing comorbidities, and extending quality life years [1]. The physical activity (PA) guidelines for older Australians [2] recommends engaging in moderate-intensity PA for at least 30 min every day. However, Australia’s health report indicates that 75% of Australians aged 65 and over do not meet these physical activity guidelines [3]. PA monitoring under free-living conditions can help determine whether the accumulation of PA meets the recommended levels, which can be used for feedback. Moreover, older people tend to increase their activity levels when monitoring PA, likely because monitoring increases motivation to achieve a PA goal [4].

An activity classification method with high accuracy is key to providing appropriate feedback to older people. Although many classification algorithms have been proposed for sensor-based activity recognition, most studies have targeted younger adults. Due to potential differences in movement features between the two age groups, the algorithms trained on young people may not be as accurate when applied to data from older people [5]. Therefore, there is a need to develop a classifier tailored for older people. As a small number of studies have specifically focused on older people using inertial sensors fixed to the trunk [6,7,8,9]. These studies utilized a combination of accelerometry, gyroscopes, and magnetometers, which were processed using neural networks, support vector machines, decision trees, or random forests to achieve accuracies of 82–93% [6,7,8,9]. These studies highlighted that accurate activity classification in older people is feasible.

However, despite high accuracy, a major downside of the abovementioned methods is that they adopted approaches which require extracting features manually based on domain knowledge and expertise. Because these hand-crafted features only contain some of the available discriminative information, their ability to differentiate activities is likely suboptimal [10]. Artificial neural networks based on deep learning techniques have been proposed for activity recognition without the need for hand-crafted feature extraction. Among all deep learning techniques, the convolutional neural network (CNN) and long short-term memory (LSTM) are commonly used for activity recognition [9,11,12]. Ignatov [13] proposed a 1D CNN model for simple physical activity classification based on triaxial acceleration data with six activity classes, and the model achieved a high accuracy of 93%. A multichannel CNN model has been designed in [11] to process data from multiple inertial measurement units (IMUs) in parallel channels. LSTM was combined with a CNN in [14], and compared to a CNN alone, with the hybrid model (DeepConvLSTM) improving the result by six percent. Due to their flexibility, neural networks with various architectures have been developed and have achieved state-of-the-art results. Unfortunately, there is no direct comparison of the performance among neural networks with different architectures for activity recognition. Moreover, how these deep learning algorithms work for an older cohort is worth investigating.

Another important consideration in the development of an activity recognition algorithm is the acceptability of the data collection method to users. To be specific, when using a wearable device to monitor PA in daily life, in order to achieve accurate insight in one’s behavior, older people must wear the device for at least four days [12], which may cause discomfort and inconvenience. On the contrary, smartphones with built-in inertial sensors can be used to gain insight into PA: while users are performing daily activities, the built-in sensors can reliably capture kinematic features [15] for activity recognition. In addition, the increasing popularity of smartphones in older people [16] makes it possible to use their own devices to perform activity monitoring. Smartphone PA monitoring does need to consider battery life, and the strain associated with certain sensing modalities such as gyroscopes. Hence, an accelerometry-based activity recognition method seems most appropriate.

This study aims to develop a classifier for accelerometry-based activity recognition in older people. Several common types activities, including lying, sitting, standing, transition, walking, walking down and walking up stairs were investigated. Among these activities, because transitioning, walking down, and walking up are closely related to the balance and the risk of falls in older people [11,13,14], we paid special attention to the classification performance of the algorithms on these activities. Four models, including a 1D CNN, a multichannel CNN, a CNN-LSTM, and a multichannel CNN-LSTM were built to explore the best architecture for this task. These models were trained on a dataset collected from older people using a smartphone. The classification and computing performance of these models was compared. Finally, the hyperparameters of the best performing model were tuned for an improved classification result, and the trained model is openly provided for further validation and use by the community.

## 2. Materials and Methods

### 2.1. Dataset Description

A dataset of free-living activities conducted by 53 older people (83.8 ± 3.8 years, 38 male and 15 female) at Neuroscience Research Australia [5] was used in this study. In this dataset, body accelerations were registered in the smartphone reference frame while participants performed a continuous series of daily-life activities, including sitting and lying on a couch, walking on level ground, standing while making a coffee, walking upstairs and downstairs (protocol in Table A1). Performance of these activities was captured with a video camera at 25 frames per second and annotated by a trained experimenter. Activities performed by the participants were annotated as lying, sitting, standing, walking, walking down, walking up, and transitions. Transitions were defined as periods during which the performed activity changed from lying to sitting, sitting to standing, standing to sitting and/or sitting to lying. Triaxial acceleration data were recorded at 100 Hz by a smartphone (Samsung Galaxy Nexus) placed in their right hand pants pocket.

### 2.2. Data Pre-Processing

#### 2.2.1. Segmentation

The continuously-sampled sensor signals were segmented using a sliding window of 2 s (i.e., 200 data points per segment) and an overlap of 1 s. Labels for each segment were assigned following the majority rule when more than one activity presented in a window.

#### 2.2.2. Training and Testing Sets

Because the algorithm developed in this study was expected to be able to cope with unknown users, a user-independent system was created. Specifically, data from 5 participants (i.e., ~10%) were randomly selected as the testing set, and the neural network was trained with data from the remaining 48 participants. The class distributions for the training and testing sets are listed in Table 1. The dataset had a skewed class distribution, and the majority classes of walking, sitting, and standing were overrepresented. This can be an issue, as models trained on an imbalanced dataset will tend to be biased towards the majority classes [17] while the free-living protocol may not provide an accurate reflection of their representation in daily life. To deal with the imbalanced dataset, a cost-sensitive learning technique was introduced: each class was mapped to a proportional weight weighting the loss function, and because the minority classes gained large weights, the importance of these classes were increased when minimizing the loss [18].

### 2.3. Implementation

The neural networks developed in this study were coded in Python (version 3.7.6). PyCharm (version 2019.3.5) [19] was used as the integrated development environment (IDE). Anaconda (version 4.8.3) [20] was set as the virtual environment for Python, and the packages installed for this study were: Numpy (version 1.18.1), Pandas (version 1.0.1), Scikit-learn (version 0.22.1), Keras (version 2.3.1), Tensorflow (version 2.1.0), Matplotlib (version 3.2.0). All analyses were carried out on a desktop with an Intel i7-8550U CPU, and 8 GB RAM.

#### 2.3.1. Neural Networks

**Figure 1:**
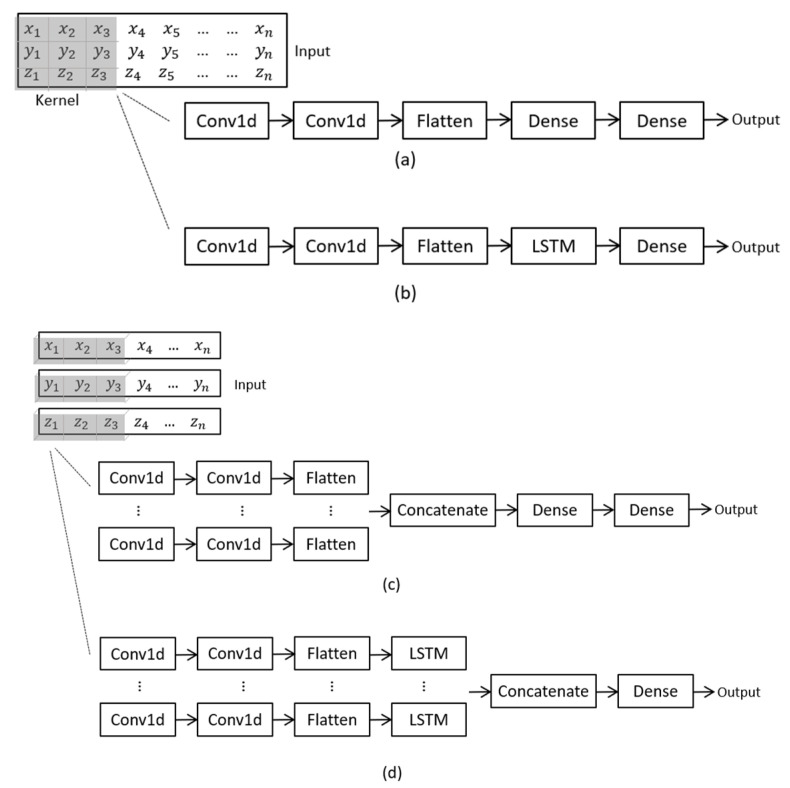
Structures of the neural networks: (**a**) 1D CNN, (**b**) CNN-LSTM, (**c**) multichannel CNN, (**d**) multichannel CNN-LSTM. x, y, z represent the triaxial accelerometer data. Abbreviations: CNN, convolutional neural network; LSTM, long short-term memory.

A 1D CNN, a multichannel 1D CNN, a CNN-LSTM hybrid model and a multichannel CNN-LSTM model (Figure 1) were tested to investigate the best structure for processing data. In developing the models, we omitted the pooling layer for a faster computation, as suggested in [21]. The “Conv1D” and “Dense” layers represent the one-dimensional convolutional layer and the fully connected layer in Keras, respectively (Figure 1). The neural networks are explained in more detail in the following sections.

##### 1D CNN

Since the 1D CNN in Keras requires a 3-dimensional input, data were shaped in a way that can be described as: input shape=(samples, steps, features), where ‘samples’ represents the total number of segments from all axes, ‘steps’ represents the sensor data at each timestamp in a segment, and ‘features’ refers to the number of axes from all the sensors used in data collection.

To compute the model, one-hot encoding was performed to convert the activity labels into dummy variables. The output of the last fully connected layer of the neural networks is the predicted label in one-hot encoding form. The hyperparameters for this model are summarized in Table A2 (see Appendix A).

##### Multichannel CNN

For the multichannel CNN model, multiple parallel channels were designed to perform convolutions on data from different axes individually. Referring to Section “1D CNN”, segments with respect to each feature in the input shape were projected to separate channels and processed by kernels with different sizes. Kernels with size of 3 and 5 were applied to the independent channels, in order to extract features from local and global fields. After the convolutional layers, the extracted feature maps for each channel were flattened and concatenated before being projected into the fully connected layer. The hyperparameters of this model are listed in Table A3 (see Appendix A).

##### CNN-LSTM Hybrid Model

The CNN-LSTM hybrid model processes input segments differently from the CNN models. To allow the LSTM to learn temporal structures from a sequence, the segment input to the CNN model was first broken into smaller sequences; the original segment (including 200 steps, which corresponds to 2 s of data sampled at 100 Hz) was divided into 10 sub-sequences, and every sub-sequence included 20 sub-steps. After the CNN extracts the local features from these sub-sequences, the LSTM interprets these features and learns the temporal relations within the entire segment. In the hybrid model, time distributed layers were used to warp the convolutional part of the model, so that each of the sub-sequences would be processed separately by the same CNN model with the same kernel weights. The shape of input for the first layer can be described as: input shape=(samples, sub_sequence, sub_steps, features), where ‘sub_sequence’ represents the number of pieces that a segment is broken into, and ‘sub_steps’ are the data points at each timestamp. The hyperparameters of the hybrid model are shown in Table A4 (see Appendix A).

##### Multichannel CNN-LSTM Hybrid Model

The multichannel CNN-LSTM model processed sub-sequences from different axes in separate channels. The input shape for this model was the same as described in Section “CNN-LSTM Hybrid Model”. Feature maps were extracted from the sub-sequences by the CNN model warped in the time distributed layers. The feature maps were subsequently flattened and projected to the LSTM layers in each channel. The outputs from each channel were merged, and the labels were predicted at the last fully connected layer. Table A5 (see Appendix A) lists the hyperparameters for this model.

#### 2.3.2. Training Algorithms

Having the structures of the neural networks defined, the training process was implemented, aiming to find a set of weights and biases that minimizes the loss during prediction. The training process involved two algorithms: forward propagation and backward propagation. Forward propagation processed the data from the first layer to the last layer. The principles and equations for forward propagation in convolutional, LSTM, and fully connected layers can be found in [17,18,22].

Every time a batch of samples completed the forward propagation, the loss was calculated using the predefined optimization and loss function to evaluate how far the predicted results were from the expected results (i.e., the prediction error). Once the loss was determined, the gradients with respect to every weight and bias were calculated backwards from the last layer to the first layer using the backpropagation algorithm. The new weights and bias were updated through the optimization algorithm in the opposite direction to the gradients, aiming to minimize the loss. The process of propagating forward and backward were repeated until the training epoch reached the predefined number (i.e., 30 epochs as all models converged before reaching this limit), representing the end of training. All the models were trained and tested 10 times and averaged results are reported.

### 2.4. Evaluation

The classification performance of the classifiers was compared by calculating the overall averaged accuracy and macro F1-score, in order to find the most accurate model. In addition, since the computational efficiency is a key factor that affect the feasibility of real-time monitoring on computationally limited platforms such as mobile applications [23,24,25], the computing performance of these models was also taken into account. As suggested in [23,25] the network size (i.e., the number of parameters of the neural networks) was measured as the criterion for consideration of installation on devices with limited resources in memory, energy, and computational capacity. The averaged training speed (i.e., the time it takes for each training epoch) and testing time were used to determine the time complexity.

## 3. Results

### 3.1. Classification and Computing Performance

The CNN-LSTM and multichannel CNN achieved a higher accuracy than the 1D CNN (74.0% and 75.5% vs. 73.4%, respectively; Table 2). When combining both multichannel and hybrid architectures in our multichannel CNN-LSTM model, the overall accuracy and macro F1-score was the best at 77.4% and 66.7%, respectively. Interestingly, the two hybrid models had a significantly smaller network size with 100,507 trainable parameters for CNN-LSTM, and 153,487 for multichannel CNN-LSTM compared to the CNN (443,619 trainable parameters) and multichannel CNN (568,279 trainable parameters), while still yielding a better classification result.

When focusing on computing time, however, the models with multichannel architecture were trained at a slower speed compared to other models. As for the testing time, the multichannel CNN-LSTM took 1.19 s to recognize all 4957 testing samples, which means that theoretically it could classify 4165 samples per second; since each sample contain data for 2 s at 100 Hz, the model seems to be sufficiently time efficient for real-time recognition, at least on the CPU used here.

**Figure 2:**
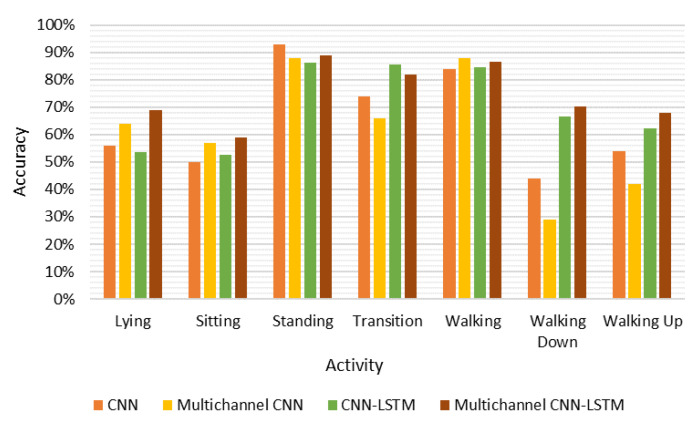
Comparison of the performance of the deep learning modes for each activity class.

Figure 2 summarizes the accuracy of each class predicted by these models. As we can see, among all these classifiers, the multichannel CNN-LSTM performed approximately equally on each class. Although larger weights were assigned to the minority classes to aid the training, walking up and down were classified with a lower accuracy irrespective of the kind of classifier used. This is also illustrated by the confusion matrices in Table A6, Table A7, Table A8 and Table A9 (see Appendix A), which illustrates that walking up and down were mostly misclassified as level walking, with false negative rates of 28.8%, 36.30%, 25.5%, and 13.3% for walking up; and 37.0%, 55.6%, 19.7%, and 16.8% for walking down for the 1D CNN, multichannel CNN, CNN-LSTM, and multichannel CNN-LSTM models, respectively. Sitting, as one of the majority class, also had a lower classification accuracy with an average of 58.3% over all the classifiers, and this activity was mostly confused with lying (see Table A6, Table A7, Table A8 and Table A9). The cause for this observation may be that these two activities did not involve motion and cause the smartphone in the pants pocket to have a similar sensor orientation, so it would be challenging to differentiate them using acceleration data. In addition, since the lying activity was always followed by the sitting activity (see Table A1 in Appendix A), the 200 data points in segments for lying might contain data for sitting, confounding the features. This notion is strengthened by the fact that the other static activity, standing, was assigned to a smaller weight but achieved a high accuracy (93.6% for 1D CNN, 88.5% for multichannel CNN, 86.3% for CNN-LSTM, and 88.9% for multichannel CNN-LSTM). This is promising because instead of standing quietly, participants actually performed daily-life tasks such as filling a glass of water, washing their hands, etc., which involved bodily motions analogue to their habitual performance in daily life.

### 3.2. Tuned Model

Because priority was given to the classification result over the computational efficiency, the multichannel CNN-LSTM was selected as the best option and tuned for a better classification performance. As suggested in [26], batch normalization layers were added to each channel and were placed after the flattening layer. After tuning the hyperparameters, the model achieved a higher overall accuracy of 81.1% (standard deviation, SD 1.5) compared to 77.4% (SD 2.8) before tuning, and the macro F1-score increased from 69.2% (SD 3.0) to 67.0% (SD 3.1). The confusion matrix of the tuned multichannel CNN-LSTM is shown in Table 3. Similar to the untuned results, most confusion occurred between lying and sitting, with false negative rates of 25.8% and 23.8%, respectively. In addition, a proportion of walking up and walking down tended to be misclassified as walking (22.5% and 23.5% for walking up and down respectively).

In terms of the computing performance of the tuned model, the model size decreased from 153,487 trainable parameters before tuning to 137,887, but the training speed was slower (18.7 s after versus 16.5 s before tuning), and there was a slight increase in the testing time (from 1.2 to 1.3 s).

## 4. Discussion

This study evaluated the classification accuracy and computational efficiency of four deep learning architectures in performing smartphone accelerometry-based activity recognition in older people. It used data of a previous study [5] that compared the generalizability of machine learning algorithms between younger and older people. Our classification results indicated that both multichannel and hybrid architectures can improve the prediction performance of the baseline CNN. When combining these two architectures, the hybrid architecture may be the dominant reason for the increased scores, as the CNN-LSTM yielded higher macro F1-scores than the multichannel CNN. Therefore, it can be inferred that the hybrid model uses the advantages of both CNN and LSTM in terms of extracting regional features within short time steps and temporal structure across a sequence. In addition, since the network size of the hybrid models was lower, there is a smaller chance of overfitting [27], which may have resulted in their higher generalizability to the validation dataset.

The multichannel architecture can generate great flexibility in designing hyperparameters. Firstly, it allows the use of different kernel sizes for separate channels, and thus enabling the network to capture both local and global patterns. Secondly, the number of kernels assigned to each channel can be different, depending on how complex the signal pattern input to the channel is. Thirdly, for the multichannel CNN-LSTM, the depth of the convolutional and LSTM layer can vary between channels, considering the trade-off between accuracy and generalization ability. The computational cost of the multichannel CNN was double that of the baseline CNN, with only a small improvement in overall accuracy (73.4% to 75.5%). Therefore, when using the CNN algorithm alone, a multichannel architecture may not be the best solution.

The developed multichannel CNN-LSTM algorithm was the most accurate approach, which seems feasible for implementation for long-term monitoring in older people. It is a smartphone-based algorithm that allows the smartphone to be set up in a way that is consistent with most people’s habits [28] (i.e., being placed in a pants pocket), and to collect data unobtrusively. Future development could consider recalibrating the reference frame of the IMU signals to the world to account for orientation changes over time [29]. In order to minimize computational cost, which has been recognized as one of the challenges in real-time activity recognition using deep learning algorithms [30], only the accelerometer was applied for activity recognition. Although classifiers can perform better when combining acceleration and gyroscope [9], additional sensors can drain battery life [31]. Despite these restrictions, the model’s classification accuracy of 81.1% is comparable to the 82–93% accuracy reported previously by studies that included more sensing modalities [6,7,8,9]. This is quite impressive given that these studies did not always split their data on an individual level or used validation, and were performed in considerably smaller sample sizes (i.e., 7–20 participants vs. our 53 participants).

While this study provides valuable insight into models for activity recognition in older people, it however also has its limitations. The main limitation of this study was the hyperparameter setting. When evaluating the four deep learning models, the batch size for each model were set to be the same, as well as the number of training epochs, for the convenience of comparison. However, there is no one-size-fits-all solution in the optimal selection of the hyperparameters, and since the hyperparameter values have a significant effect on the performance of the classifiers [32], changing these values could have led to different results. In addition, the computational cost depends on the device used for implementation, and the cost may vary when using a different device. Further investigation is required to understand how well the multichannel CNN-LSTM algorithm performs on smartphones in terms of computational efficiency.

Another issue was related to the smartphone use and placement. Since the smartphone was placed in the pants pocket, our results may not generalize to other sensor placements which could be addressed in future studies. In addition, smartphones are used for more than activity tracking and may not always be worn in one’s pocket. Future studies could consider development of an algorithm to detect whether the phone is in use or worn in the pants pocket. Moreover, the pants pocket placement may not be effective to capture upper body movements or arm movements, which are common during daily activities such as cooking and vacuuming. As a result, equipping a sensor such as a smartwatch on upper limbs may be essential for complex activities recognition. Future research should consider regrouping activities and adding in additional sensors or devices such as smartwatches to improve classification performance. For instance, the static activities that do not contribute to the accumulation of the PA level, such as lying and sitting, may be grouped into the resting activity as proposed by Khan et al. [33] for an improved performance. Furthermore, as indicated by Voicu et al. [34], walking upstairs and downstairs are easily confused with levelled walking, therefore collecting more data with, e.g., barometric pressure sensors during these activities might enable the classifier to learn more discriminative features.

Our trained algorithm is freely available for clinical and research applications (osf.io/47vx6); however, generalizability needs to be established. With ageing, functional capacity declines [35] and gait and balance disorders arise [36]. As a result, the model that is trained on data from a finite group of participants may not suit a more heterogeneous group. We used data of 53 older people, which was considerably more than the 1 to 23 of participants in previous studies [6,7,8,9]. In addition, our data included both non-frail and frail participants. As reported by Del Rosario et al. [5], the generalizability of the multichannel CNN-LSTM was assumed to be better than the algorithms that were trained on a smaller data set. Another way to manage heterogeneity is to personalize the classifier. Personalized activity recognition using transfer learning technique was proven to be valid by Tahavori et al. [37]. This technique achieved a high testing accuracy by using two training steps: a CNN was first pre-trained on the training participants, and then the output layer was retrained with only three labelled samples from the new participants. Because personalized activity recognition requires retraining the algorithm for every user, it may be better to choose a robust algorithm with good generalizability for most of the non-frail users, and implement the personalized technique for users who have abnormal gait and need particular attention.

## 5. Conclusions

CNNs and LSTMs have been widely used in activity recognition in younger adults but their performance in older people has been understudied. In order to implement activity recognition through ubiquitous technologies such as smartphones and smartwatches in older and frailer population groups, it is important that activity recognition can be tailored to achieve a more correct classification. We compared the accuracy and computational efficiency of four different deep learning algorithms for smartphone-based activity recognition in older people. User-independent models, including a 1D CNN, a multichannel CNN, a CNN-LSTM, and a multichannel CNN-LSTM were built and trained on an imbalanced dataset with a cost-sensitive learning technique. Based on the comparison, the multichannel CNN-LSTM achieved the best classification result with an acceptable computational cost. Therefore, it is feasible to use deep learning algorithms for activity recognition using smartphone accelerometry data in older people, and the multichannel CNN-LSTM appears to be a good solution weighting both accuracy and computational efficiency.

## Figures and Tables

**Table 1:** Class distributions for the training and testing sets.

Activity	Training Set (N = 43)		Testing Set (N = 5)	
	Segments (2 s Each)	Percentage of Total (%)	Segments (2 s Each)	Percentage of Total (%)
Lying	1576	5.0	243	4.9
Sitting	2007	26.7	1365	27.5
Standing	2480	18.0	755	15.2
Transition	1907	6.1	246	5.0
Walking	12,405	39.7	2158	43.5
Walking Down	681	2.2	90	1.8
Walking Up	748	2.4	100	2.0

**Table 2:** Classification and computing performance of the deep learning models (averaged results for 10 runs with standard deviation).

Model	1D CNN	Multichannel CNN	CNN-LSTM	Multichannel CNN-LSTM
Trainable parameters	443,619	568,279	100,507	153,487
Training speed (s/epoch)	8.69	16.95	10.42	16.63
Testing time (s)	0.39	0.83	0.68	1.19
Accuracy (%)	73.4 (3.8)	75.5 (3.4)	74.0 (2.3)	77.4 (2.8)
Macro F1-score (%)	62.5 (2.3)	59.3 (2.2)	63.4 (2.3)	66.7 (3.1)

**Table 3:** Confusion matrix for the tuned multichannel CNN-LSTM (in percentage; darker blue indicates higher values).

Predicted								
		Lying	Sitting	Standing	Transition	Walking	Walking Down	Walking Up
**Actual**	Lying	67.0	25.8	2.5	3.7	0.2	0.4	0.4
	Sitting	23.8	70.7	3.4	1.8	0.2	0.1	0.0
	Standing	0.1	3.7	88.4	0.9	6.3	0.5	0.2
	Transition	1.9	5.4	6.1	78.2	4.9	1.6	2.0
	Walking	0.0	0.0	5.6	1.2	88.7	2.6	2.0
	Walking Down	0.0	0.0	3.7	5.0	23.5	65.7	2.0
	Walking Up	0.0	0.0	0.7	5.7	22.5	2.5	68.7

## Appendix A

**Table A1:** Data collection protocol for the free-living activities.

Task	Description
1	In a room, stand in front of a couch, sit on the couch, lie down, and repeat in reverse; walk to a window and look outside; sit on a chair; switch off a wall power outlet and turn off the light before leaving the room.
2	Walk to the kitchen, fill a cup of water, take a sit at a table on one side, and drink the water; empty the cup at the kitchen sink, and dry hands.
3	Walk to the elevator, and ride the elevator up to Level 2; walk along the hallway, stand and wait; walk back the elevator, and ride the elevator up to Level 3; walk along the hallway, and take a sit on a couch; walk back to elevator, and go down to the ground floor.
4	Walk to a short stairs, and walk upstairs; walk to a long stairs and walk upstairs; walk to a room and take a sit on a chair; walk back to the long stairs and walk downstairs; walk to the short stairs and walk downstairs.
5	Walk to an armchair and take a sit; walk to a couch in a room, lie down, and sit up.

**Table A2:** Hyperparameter values for the 1D CNN.

	Hyperparameter		Value
Architecture	Number of Conv1d layers		2
	Number of kernels for each conv1d		35
	Kernel size		3
	Stride		1
	Number of neurons	First dense layer	64
		Second dense layer	7
Training	Optimization algorithm		Adadelta
	Learning rate		0.001
	Batch size		128
	Training epochs		30

**Table A3:** Hyperparameter values for the multichannel CNN.

	Hyperparameter		Value
Architecture	Number of Conv1d layers per channel		2
	Number of kernels	Channel 1	32
		Channel 2	20
		Channel 3	20
	Kernel size	Channel 1	3
		Channel 2	3
		Channel 3	5
	Stride		1
	Number of neurons	First dense layer	40
		Second dense layer	7
Training	Optimisation algorithm		Adadelta
	Learning rate		0.001
	Batch size		128
	Training epoch		30

**Table A4:** Hyperparameter values for the CNN-LSTM.

	Hyperparameter		Value
Architecture	Number of Conv1d layers		2
	Number of kernels for each conv1d		35
	Kernel size		3
	Stride		1
	Number of neurons	LSTM	40
		Dense layer	7
Training	Optimization algorithm		Adadelta
	Learning rate		0.001
	Batch size		128
	Training epoch		30

**Table A5:** Hyperparameter values for the multichannel CNN-LSTM.

	Hyperparameter		Value
Architecture	Number of Conv1d layers per channel		2
	Number of kernels	Channel 1	35
		Channel 2	20
		Channel 3	20
	Kernel size	Channel 1	3
		Channel 2	3
		Channel 3	5
	Stride		1
	Neurons for LSTM	Channel 1	30
		Channel 2	30
		Channel 3	30
Training	Optimization algorithm		Adadelta
	Learning rate		0.001
	Batch size		128
	Epochs		30

**Table A6:** Confusion matrix for the 1D CNN (in percentages, averaged over 10 runs).

Predicted								
		Lying	Sitting	Standing	Transition	Walking	Walking Down	Walking Up
Actual	Lying	56.38	36.76	0.00	6.72	0.14	0.00	0.00
	Sitting	27.61	50.34	17.91	3.82	0.23	0.02	0.06
	Standing	0.00	0.57	93.60	0.73	5.01	0.07	0.02
	Transition	2.51	6.30	8.88	74.05	5.76	1.49	1.02
	Walking	0.00	0.22	11.12	2.06	84.80	1.15	0.65
	Walking Down	0.00	1.48	7.96	2.04	37.04	44.44	7.04
	Walking Up	0.00	0.00	4.17	8.33	28.83	4.00	54.67

**Table A7:** Confusion matrix for the multichannel CNN (in percentages, averaged over 10 runs).

Predicted								
		Lying	Sitting	Standing	Transition	Walking	Walking Down	Walking Up
Actual	Lying	64.49	29.05	0.78	4.86	0.12	0.37	0.33
	Sitting	31.48	57.44	9.22	1.30	0.19	0.18	0.19
	Standing	0.01	2.15	88.54	1.22	7.54	0.37	0.17
	Transition	3.29	7.76	8.66	66.18	7.93	2.60	3.58
	Walking	0.00	0.08	7.15	1.33	88.18	1.36	1.90
	Walking Down	0.00	0.33	6.11	4.67	55.56	29.44	3.89
	Walking Up	0.00	0.20	5.30	12.10	36.30	3.50	42.60

**Table A8:** Confusion matrix for the CNN-LSTM (in percentages, averaged over 10 runs).

Predicted								
		Lying	Sitting	Standing	Transition	Walking	Walking Down	Walking Up
Actual	Lying	53.50	38.07	0.31	7.61	0.00	0.31	0.21
	Sitting	27.62	52.75	16.15	3.17	0.15	0.11	0.05
	Standing	0.00	0.43	86.32	7.52	5.50	0.20	0.03
	Transition	1.52	2.34	4.88	85.67	2.95	0.61	2.03
	Walking	0.00	0.07	4.77	6.53	84.43	1.67	2.53
	Walking Down	0.00	0.83	2.50	6.11	19.72	66.67	4.17
	Walking Up	0.00	0.00	2.25	9.25	25.50	0.75	62.25

**Table A9:** Confusion matrix for the multichannel CNN-LSTM (in percentages, averaged over 10 runs).

Predicted								
		Lying	Sitting	Standing	Transition	Walking	Walking Down	Walking Up
Actual	Lying	69.09	23.50	1.44	4.57	0.00	0.91	0.49
	Sitting	33.45	59.14	5.19	1.74	0.11	0.35	0.02
	Standing	0.40	3.05	88.91	0.98	5.06	0.77	0.83
	Transition	2.85	3.94	4.55	82.24	2.20	2.32	1.91
	Walking	0.00	0.00	6.69	1.28	86.00	2.58	3.45
	Walking Down	0.00	0.11	2.67	8.33	16.78	70.11	2.00
	Walking Up	0.00	0.00	1.40	10.10	13.30	6.80	68.40

## References

[1] Taylor D. (2014). Physical activity is medicine for older adults Postgrad. Med. J..

[2] Australian Government Department of Health Population Health Australia’s Physical Activity and Sedentary Behaviour Guidelines and the Australian 24-Hour Movement Guidelines. https://www1.health.gov.au/internet/main/publishing.nsf/Content/health-pubhlth-strateg-phys-act-guidelines.

[3] Australian Institute of Health Welfare (2018). Insufficient Physical Activity. Australia’s Health 2018.

[4] Gaz D.V., Rieck T.M., Peterson N.W., Sozen H. (2016). Activity Tracking and Improved Health Outcomes. Fitness Medicine.

[5] Del Rosario M.B., Wang K., Wang J., Liu Y., Brodie M., Delbaere K., Lovell N.H., Lord S.R., Redmond S.J. (2014). A comparison of activity classification in younger and older cohorts using a smartphone. Physiol. Meas..

[6] Awais M., Chiari L., Ihlen E.A.F., Helbostad J.L., Palmerini L. (2018). Physical Activity Classification for Elderly People in Free-Living Conditions. IEEE J. Biomed. Heal. Inform..

[7] Papagiannaki A., Zacharaki E., Kalouris G., Kalogiannis S., Deltouzos K., Ellul J., Megalooikonomou V. (2019). Recognizing Physical Activity of Older People from Wearable Sensors and Inconsistent Data. Sensors.

[8] Sansrimahachai W., Toahchoodee M. Mobile-phone based immobility tracking system for elderly care. Proceedings of the 2016 IEEE Region 10 Conference (TENCON).

[9] Tahavori F., Stack E., Agarwal V., Burnett M., Ashburn A., Hoseinitabatabaei S.A., Harwin W. Physical activity recognition of elderly people and people with parkinson’s (PwP) during standard mobility tests using wearable sensors. Proceedings of the 2017 International Smart Cities Conference (ISC2).

[10] Wang J., Chen Y., Hao S., Peng X., Hu L. (2019). Deep learning for sensor-based activity recognition: A survey. Pattern Recognit. Lett..

[11] Rueda F.M., Grzeszick R., Fink G.A., Feldhorst S., Hompel M.T. (2018). Convolutional Neural Networks for Human Activity Recognition Using Body-Worn Sensors. Informatics.

[12] Van Schooten K.S., Rispens S.M., Elders P.J., Lips P., Van Dieën J.H., Pijnappels M. (2015). Assessing Physical Activity in Older Adults: Required Days of Trunk Accelerometer Measurements for Reliable Estimation. J. Aging Phys. Act..

[13] Ignatov A. (2018). Real-time human activity recognition from accelerometer data using Convolutional Neural Networks. Appl. Soft Comput..

[14] Ordóñez F.J., Roggen D. (2016). Deep Convolutional and LSTM Recurrent Neural Networks for Multimodal Wearable Activity Recognition. Sensors.

[15] Jain A., Kanhangad V. (2018). Human Activity Classification in Smartphones Using Accelerometer and Gyroscope Sensors. IEEE Sensors J..

[16] Klimova B., Maresova P., Park J.J., Jin H., Jeong Y.-S., Khan M.K. (2016). Elderly People and Their Attitude Towards Mobile Phones and Their Applications—A Review Study. Advanced Multimedia and Ubiquitous Engineering.

[17] Brownlee J. A gentle Introduction to Imbalanced Classification, Machine Learning Mastery, 22 December 2019. https://machinelearningmastery.com/what-is-imbalanced-classification/.

[18] López V., Fernández A., Moreno-Torres J.G., Herrera F. (2012). Analysis of preprocessing vs. cost-sensitive learning for imbalanced classification. Open problems on intrinsic data characteristics. Expert Syst. Appl..

[19] JetBrains, Pycharm. https://www.jetbrains.com/pycharm/.

[20] Anaconda, Inc. Anaconda Software Distribution. https://anaconda.com.

[21] Gholamrezaii M., Almodarresi S.M.T. Human Activity Recognition Using 2D Convolutional Neural Networks. Proceedings of the 2019 27th Iranian Conference on Electrical Engineering (ICEE).

[22] Ejupi A., Brodie M., Lord S.R., Annegarn J., Redmond S.J., Delbaere K. (2017). Wavelet-Based Sit-To-Stand Detection and Assessment of Fall Risk in Older People Using a Wearable Pendant Device. IEEE Trans. Biomed. Eng..

[23] Howard A.G., Zhu M., Chen B., Kalenichenko D., Wang W., Weyand T., Andreetto M., Adam H. (2017). MobileNets: Efficient convolutional neural networks for mobile vision applications. arXiv.

[24] Jeon Y., Kim J., Bengio S., Wallach H., Larochelle H., Grauman K., Cesa-Bianchi N., Garnett R. (2018). Constructing fast network through deconstruction of convolution. Advances in Neural Information Processing Systems 31.

[25] Ignatov A., Timofte R., Chou W., Wang K., Wu M., Hartley T., Van Gool L. (2018). AI Benchmark: Running Deep Neural Networks on Android Smartphones. arXiv.

[26] Garbin C., Zhu X., Marques O. (2020). Dropout vs. batch normalization: An empirical study of their impact to deep learning. Tools Appl..

[27] Brownlee J. (2018). How to Avoid Overfitting in Deep Learning Neural Eetworks, Machine Learning Mastery. https://machinelearningmastery.com/introduction-to-regularization-to-reduce-overfitting-and-improve-generalization-error/.

[28] Sun L., Zhang D., Li B., Guo B., Li S., Yu Z., Liscano R., Chen G., Zhang D., Zhou X. (2010). Activity recognition on an accelerometer embedded mobile phone with varying positions and orientations. Ubiquitous Intelligence and Computing.

[29] Del Rosario M.B., Lovell N.H., Redmond S.J. (2016). Quaternion-Based Complementary Filter for Attitude Determination of a Smartphone. IEEE Sens. J..

[30] Chen K., Zhang D., Yao L., Guo B., Yu Z., Liu Y. (2020). Deep learning for sensor-based human activity recognition: Overview, challenges and opportunities. arXiv.

[31] Mortazavi B.J., Nemati E., Vanderwall K., Flores-Rodriguez H.G., Cai J.Y.J., Lucier J., Naeim A., Sarrafzadeh M. (2015). Can Smartwatches Replace Smartphones for Posture Tracking?. Sensors.

[32] Wu J., Chen X.-Y., Zhang H., Xiong L.-D., Lei H., Deng S.-H. (2019). Hyperparameter optimization for machine learning models based on Bayesian optimization. J. Electron. Sci. Technol..

[33] Khan A.M., Lee Y.-K., Lee S., Kim T.-S. (2010). Accelerometer’s position independent physical activity recognition system for long-term activity monitoring in the elderly. Med. Biol. Eng. Comput..

[34] Voicu R.-A., Dobre C., Băjenaru L., Ciobanu R.-I. (2019). Human Physical Activity Recognition Using Smartphone Sensors. Sensors.

[35] Tuna H.D., Özcan A., Malkoc M., Aksakoglu G. (2009). Effect of age and physical activity level on functional fitness in older adults. Eur. Rev. Aging Phys. Act..

[36] Gait and Balance Disorders in Older Adults—American Family Physician. https://www.aafp.org/afp/2010/0701/p61.html.

[37] Rokni S.A., Nourollahi M., Ghasemzadeh H. (2018). Personalized human activity recognition using convolutional neural networks. arXiv.

